# Correction: Indirect fabrication of versatile 3D microfluidic device by a rotating plate combined 3D printing system

**DOI:** 10.1039/c8ra90091d

**Published:** 2018-12-05

**Authors:** Dong-Heon Ha, Dong-Hyeon Ko, Jin-Oh Kim, Do Jin Im, Byoung Soo Kim, Soo-Young Park, Steve Park, Dong-Pyo Kim, Dong-Woo Cho

**Affiliations:** Department of Mechanical Engineering, Pohang University of Science and Technology (POSTECH) Pohang South Korea dwcho@postech.ac.kr; Department of Chemical Engineering, Pohang University of Science and Technology (POSTECH) Pohang South Korea dpkim@postech.ac.kr; Department of Materials Science and Engineering, Korea Advanced Institute of Science and Technology (KAIST) Daejeon Korea; Department of Chemical Engineering, Pukyong National University Busan South Korea; Department of Polymer Science and Engineering, Kyungpook National University Daegu South Korea

## Abstract

Correction for ‘Indirect fabrication of versatile 3D microfluidic device by a rotating plate combined 3D printing system’ by Dong-Heon Ha *et al.*, *RSC Adv.*, 2018, **8**, 37693–37699.

The authors regret that the name of one of the authors (Jin-Oh Kim) was shown incorrectly in the original article. In addition, the author contributions were incorrectly given. The corrected author list and contributions are as shown above.

The Royal Society of Chemistry apologises for these errors and any consequent inconvenience to authors and readers.

## Supplementary Material

